# Spheres of uncertainty: A phenomenological inquiry into healthcare practice surrounding the care for people with relapsing-remitting multiple sclerosis

**DOI:** 10.1016/j.qrmh.2025.100031

**Published:** 2025-11-19

**Authors:** Eva C. van Reenen, Alistair R. Niemeijer, Leo H. Visser, Janet W.K. de Beukelaar, Bob W. van Oosten, Stephan T.F.M. Frequin, Erwin L.J. Hoogervorst, Inge A.M. van Nistelrooij

**Affiliations:** a Care Ethics, University of Humanistic Studies, Kromme Nieuwegracht 29, 3512 HD Utrecht, The Netherlands; b Neurology, Elisabeth-TweeSteden Hospital, Hilvarenbeekseweg 60, 5022 GC Tilburg, The Netherlands; c Neurology, Albert Schweitzer Hospital, Albert Schweitzerplaats 25, 3318 AT Dordrecht, The Netherlands; d Neurology, Amsterdam UMC - VU Medical Centre, De Boelelaan 1117 / 1118, 1081 HV Amsterdam, The Netherlands; e Neurology, St. Antonius Hospital, Soestwetering 1, 3543 AZ Utrecht, The Netherlands

**Keywords:** Uncertainty, Multiple sclerosis, Qualitative research, Phenomenology, Embodied enquiry, Shadowing

## Abstract

**Background:**

Uncertainty is pervasive in healthcare and permeates every clinical encounter between patients and medical professionals. Patients with specifically uncertain diagnoses or treatments such as multiple sclerosis (MS) are more likely to respond negatively to a lack of clear information. Research into MS has a narrow focus on scientific issues of uncertainty, such as causal explanations or treatment recommendations. Inquiry into the interplay between various dimensions, contexts, and subjects of uncertainty in a relational practice and institutional context, is scarce. The objective of this research is to investigate the phenomenon of uncertainty as it appears in hospital practice surrounding outpatient care for people with relapsing-remitting multiple sclerosis (RRMS).

**Methods:**

This study followed a phenomenological research design, inspired by the work of Les Todres on embodied enquiry. Fifteen people with a recent (less than one year) diagnosis of RRMS were included and prospectively shadowed during hospital appointments over the course of two years.

**Results:**

The phenomenon of uncertainty is captured as occurring in four different spheres: 1) precarious spaces, 2) elusive technology, 3) hidden expectations, and 4) unsure communication. The image of spheres points to their varied and sometimes opposing (sur)faces when rotated around their axis. The spheres can increase or decrease a sense of doubt, confusion, restlessness, or anxiety in both patients and healthcare providers.

**Conclusion:**

The four different spheres never seem to fully surface, making uncertainty a masked phenomenon. The findings imply a need for an “unveiling” of uncertainty through 1) examining and debating the course of action at the outpatient clinic, 2) reconsidering the promises and perils of technology, and 3) through metacommunication with patients.

## Highlights

• Uncertainty is pervasive in healthcare, especially in relapsing-remitting MS.
• Patients and healthcare providers navigate multiple ‘spheres’ of uncertainty.
• Technology, such as MRI scans, can both reduce and increase uncertainty.
• Implicit expectations and communication challenges shape the care experience.
• Greater awareness and open discussion can help address uncertainty in healthcare.

Uncertainty is pervasive in healthcare and permeates every clinical encounter between patients and medical professionals (Han et al., 2021, Hillen et al., 2017, Kalke et al., 2021). Defined as the awareness of ignorance (Han et al., 2011), the unknowns in healthcare are abundant: whether a specific diagnosis can be established, how a condition will develop, what treatment a patient will benefit from, etc. (Hillen et al., 2017). Uncertainty affects both patients and physicians and may lead to adverse emotional and behavioral effects such as anxiety, vulnerability, and indecision (Han et al., 2021, Kalke et al., 2021). In an attempt to manage these effects, both providers and recipients of care show a tendency towards reducing uncertainty through information seeking (Han et al., 2021). Not all forms of uncertainty, however, can be resolved this way (Han et al., 2011). Furthermore, in the rapidly evolving field of medicine, technological, and scientific advancements complicate the quest for clear-cut answers (Kalke et al., 2021, Simpkin and Schwartzstein, 2016).

Research shows that patients with inherently uncertain diagnoses, prognoses, or effects of treatment, experience higher levels of “intolerance of uncertainty”—negative responses to a lack of clear information—than populations with clear diagnoses and treatment plans (Yang et al., 2021). One can imagine, for example, the medical follow-up for a broken leg to be more straightforward than for cancer or chronic diseases such as multiple sclerosis (MS), where coping with uncertainty proves a major challenge (Alschuler & Beier, 2015). Relapsing-remitting MS (RRMS)—one of the phenotypes affecting the majority (about 80–85 %) of MS patients—is characterized by periods of relapses followed by complete or partial recovery (Dobson and Giovannoni, 2019, Lublin et al., 2014, Thompson et al., 2018a). The unpredictable occurrence and timing of these relapses, limited diagnostic tests, and variable drug efficacy all complicate communication between patients and medical providers (Price et al., 2021).

In order to better understand and cope with uncertainty, Han and colleagues (2011) argue for a shared concept of uncertainty. They integrate existing theories on uncertainty into a comprehensive three-dimensional taxonomy that characterizes uncertainty in healthcare through its (1) sources (probability, ambiguity, and complexity), (2) issues (scientific, practical, and personal), and (3) locus (patient or healthcare provider). The multidimensional character of the taxonomy aims to expand the focus of research and clinical management to more than a single source or issue of uncertainty (Han et al., 2011). When applied in practice, however, the different dimensions still seem to be employed as separate entities. The theoretic distinction between reducible uncertainty (stemming from complexity) and irreducible uncertainty (stemming from probability) may not hold or prove universally helpful in real-world scenarios, which are often complex and rarely clear-cut (Blumenthal, 2010, p. 19–21; Buetow, 2011).

Research into MS exhibits a similarly narrow focus on particular, mostly scientific, issues of uncertainty such as finding causal explanations (Bjornevik et al., 2022) or attempting to improve treatment recommendations (Harding et al., 2019). Inquiry into the personal and practical issues of uncertainty is scarce. More importantly, the interplay between the various dimensions, contexts, and subjects of uncertainty have received insufficient investigatory consideration. A previous study into the experiences of people with RRMS with uncertainty shows that uncertainty is a multifaceted experience, fueled by the physical, mental, and social sequelae of the disease (Van Reenen et al., 2025). Both internal and external processes and events can expose ambivalence, suggesting a complex interaction between cognition and context.

It would be epistemologically naïve to ignore the embeddedness of the loci of uncertainty—patient and physician—in a relational practice and institutional context (Leget et al., 2019). Recognizing this interrelation highlights the need to include multiple facets of uncertainty discussed above, as they interact with professional relationships and organizational settings. Because of this complexity, a qualitative, phenomenological approach provides a more holistic inquiry into uncertainty (Finlay, 2011, p. 21). It is through the lifeworld, or “a humanly relational world, full of meanings” (Galvin & Todres, 2013, p. 25) that a phenomenon (an event, object, situation, process) can be known (Finlay, 2011, p. 16). To contribute to the understanding and management of uncertainty, this study aimed to investigate the phenomenon of uncertainty as it appears in hospital practice surrounding outpatient care for people with RRMS.

## Methods

### Study design

This study followed a longitudinal, phenomenological design. This allowed for repeated data collection during events of interest, minimizing recall bias, and following change over time (Caruana et al., 2015). The aim of phenomenology is to capture embodied lived experience, or the world as we directly experience it (Finlay, 2011, p. 15–16). Phenomenologists such as Merleau-Ponty claim the body is essential to understanding the human condition (Finlay, 2011, p. 36). Perception arises from our bodily engagement with the world, rather than solely through the senses and interpretation by the brain (Finlay, 2011, p. 21).

This study is inspired by the work of Les Todres (2007) on embodied enquiry, which emphasizes the lived body as both a vehicle for and foundation of understanding (Todres, 2007, p. 21). Embodied knowing refers not only to intellectual reflection, but to knowledge that is inherently sensory, affective, and situated. For example, during a hospital consultation, the embodied experience may include the sensation of the chair beneath the patient, the tone and pace of the physician’s voice, and the physical proximity between the two, all of which can shape meaning making beyond the spoken words. This type of qualitative research seeks to carry forward meaning through textured bodily experience which is “always on the way” (Todres, 2007, p. 29) and aims to retain the richness and texture of individual experiences when formulating a general description (Todres, 2007, p. 7–9). Rather than maintaining the strict dichotomy between descriptive and interpretive phenomenology, this approach attempts to harmonize analytical rigor with evocative expression in order to convey the lived quality of the phenomenon under study (Todres, 2007, p. 45).

### Recruitment and participants

Participants were recruited among four Dutch hospitals with specialized MS centers. Inclusion criteria included a recent (less than one year) diagnosis of RRMS based on the McDonald criteria (Thompson et al., 2018b), being at least 18 years of age, and in the process of deciding on or starting with treatment. Possible participants were approached through an online call and through the recruiting hospital neurologists and specialized MS-nurses. Prospective participants reached out to the principal investigator (EvR) or vice versa and were provided with an information letter.

In phenomenological research, the sample size is determined by the scope and depth of the phenomenon under study and is considered sufficient once a rich description is obtained (Dahlberg et al., 2008, p. 175–176). Seventeen participants were recruited in total, 15 of whom were included in this study (see Table 1). The excluded two participants yielded no empirical data, as no hospital appointments were shadowed. Four of the 15 remaining participants were male and 11 were female, ranging in ages from 25 to 60 years old. One of the participants (P12) was initially included with a diagnosis of RRMS, but the diagnosis was later changed to clinically isolated syndrome (CIS)—a precursor to MS. As receiving an MS diagnosis is an profound and meaningful experience (Solari et al., 2007), uncertainty about or reversal of the initial diagnosis is relevant to MS care. The research team therefore decided to maintain the inclusion.

**Table 1:** Characteristics of individual participants

	Name (pseudonym)	Self-identified gender	Age (years)	Appointments shadowed
P01	Michael	Male	60	*1. Consultation - Neurologist* *2. MRI - Neurologist* *3. MRI - Neurologist*
P02	Sophie	Female	38	*1. Consultation - Neurologist* *2. MRI - Neurologist*
P03	Patricia	Female	60	*1. MRI - Neurologist* *2. Consultation - Neurologist*
P04	Max	Male	26	*1. Consultation - Neurologist*
P05	Nicole	Female	32	*1. Consultation - MS nurse* *2. MRI - Neurologist*
P06	Alice	Female	28	*1. Consultation - Neurologist* *2. MRI - Neurologist*
P07	Sarah	Female	38	*1. Consultation - Neurologist* *2. Consultation - Neurologist* *3. MRI - Neurologist*
P08	Audrey	Female	30	*1. Medication - MS nurse*
P10	Dylan	Male	27	*1. Consultation - Neurologist* *2. MRI - Neurologist* *3. MRI - Neurologist*
P11	Abigail	Female	40	*1. Consultation - Neurologist*
P12	Ava	Female	25	*1. Medication - MS nurse* *2. Consultation - Neurologist* *3. MRI - Neurologist* *4. MRI - Neurologist* *5. Consultation - Neurologist*
P14	Kate	Female	43	*1. Medication - MS nurse* *2. Consultation - Neurologist* *3. MRI - Neurologist*
P15	Margaret	Female	58	*1. MRI - Neurologist* *2. Consultation - Neurologist*
P16	Wendy	Female	47	*1. MRI - Neurologist* *2. Consultation - Neurologist*
P17	Matthew	Male	29	*1. Consultation - Neurologist* *2. MRI - Neurologist* *3. Consultation - Neurologist* *4. Consultation - Neurologist*

### Data collection

The Medical Ethical Review Committee Brabant (The Netherlands), the Ethical Review Committee of the University of Humanistic Studies Utrecht (The Netherlands), and the Review Boards of the four participating hospitals checked and confirmed participants’ rights, safety, and well-being. The 15 participants were followed over the course of two years and shadowed by a member of the research team (EvR)^2^ during hospital appointments. Shadowing is a form of non-participant observation where a researcher observes, but does not participate (Johnson, 2014, p. 30). This method allows for the collection of data on behavior and actions and on the purposes, intentions, and lived meanings through which individuals understand and experience them (McDonald, 2005). Shadowing is well-suited for phenomenological research, as it enables the researcher to be physically present in the lifeworld of participants and attuned to the experiential meanings that arise in practice (Van der Meide et al., 2013).

Periodic check-ups and occassional impromptu appointments—scheduled according to participant availability and treatment follow-ups—were shadowed. After diagnosis, patients typically have several appointments to discuss treatment and evaluate MRI results (Dutch Federation of Neurologists, 2023). We regularly contacted participants to inquire about newly scheduled medical appointments. At times, participants would inform us themselves. We would always attempt to clear our schedules and be present. A few appointments were missed due to prior engagements or personal circumstances on our part or due to miscommunication. In total, 39 appointments were shadowed, three of which were excluded from analysis because they lacked either the relevant context (the outpatient clinic) or the presence of both relevant parties (patient and healthcare provider). These excluded cases involved either a video consultation with the neurologist while we were at the participant’s home or a phone call from the hospital in which we were with the healthcare provider in the consultation room and the participant responded from home.

We met participants, sometimes accompanied by their partner, at the neurology clinic. In the waiting room, a variety of topics would be discussed, ranging from the period leading up to the appointment to anticipated questions for the neurologist, health issues, work, holidays, or something happening in the waiting room. During appointments, we positioned ourselves apart from both the participant and the healthcare professional, usually at the edge of the room against a wall, remaining as silent and unobtrusive as possible. Following the consultation, we would leave the room with the participant and ask whether they had the time and headspace for a brief reflection on the hospital visit. These informal conversations took place while walking toward the hospital exit, standing in a hallway, or sitting in the waiting room or the hospital restaurant. Shadowing lasted anywhere from 30 minutes to three hours, depending on the length of the appointment and any subsequent conversations. During that time, we documented in detail what we saw, heard, felt, and thought. This typically yielded several pages of handwritten field notes in a notebook, capturing as closely as possible the exact words of the observed individuals in short sentences. As soon as both parties parted ways, we edited and expanded our notes into a comprehensive, chronological report. When it was not possible to draw up a report immediately, we made an audio recording of additional impressions to be elaborated at a later stage.

### Data analysis

Phenomenological analysis is iterative, involving repeated reading of the collected material, lingering on selected passages, and formulating evolving understanding (Finlay, 2011, p. 228–229). In line with this approach, no formal coding was conducted. Instead, we began analysis during data collection, grouping significant events thematically (Finlay, 2011, p. 234). Reflexive analysis considered “how the researcher, the context and the relationship between researcher and participants have influenced the data gathered and findings” (Finlay, 2011, p. 239). Careful attention was paid to distinguishing between participants’ perspectives and our own assumptions.

This process was supported by ongoing discussions with the entire research team (EvR, AN, LV, IvN), and informed by our reflexive logbooks in which embodied responses were recorded during and after each observation. Interpretations were grounded in the situational context of the observations, with implicit meanings explored through re-reading of field notes to ensure contextual accuracy (Todres, 2007, p. 40). The analysis was not limited to patients’ perspectives. It also included the perspectives of healthcare professionals, captured through shadowing, as well as the relational dynamics between them—elements inseparable from our embodied, situated knowing, which, through lived presence and reflective awareness, opens up insight into the phenomenon (Todres, 2007, p. 21).

To bring the knowing and experiencing body into the text, sensory and contextual details were integrated, noting bodily expressions, spatial arrangements, and other non-verbal elements that shaped the encounters. Evocative language was used to engage readers with the phenomenon of uncertainty, capturing its complexity and ambivalence (Finlay, 2011, p. 232; Galvin & Todres, 2013, p. 158; Todres, 2007, p. 41). Data were continuously reworked in light of new insights, until the findings were judged sufficiently rich and resonant to convey the lived meaning of the phenomenon (Galvin & Todres, 2013, p. 167).

### Ethical considerations

After IRB approval was obtained, participants received an information letter and were briefed about the study. Pseudonimity was ensured by assigning a number to all participants and removing identifying information from field notes and reports. Data were stored in a secured project file and access was restricted to members of the research team. Informed consent was obtained at the first meeting, with participants re-confirming their willingness before and during each appointment. We notified the involved neurologist or MS nurse of our presence in advance.

Shadowing can lead to “unclear situations that arise in the field in which there are conflicting standards” and that have not or could not have been addressed beforehand because such situations are context-specific and often unpredictable (Johnson, 2014, p. 24). One such challenge was balancing rapport-building with participants and keeping the activities and conversations directed toward the practice of interest (Duncombe and Jessop, 2014, Johnson, 2014, p. 32). At times, the conversations in the waiting room between researcher and participant resembled those between acquaintances or friends: talking about plans for the weekend or making jokes about something that happened in the hospital. Fostering an “ethic of friendship,” or even for friendships to develop in qualitative research, is not uncommon and may even benefit both researcher and participant (Tillmann-Healy, 2003). Researchers may reach a deeper level of understanding and depth of experience, and participants can feel heard, known, and understood (Tillmann-Healy, 2003). To safeguard this dynamic, participants were clearly informed—both orally and through an information sheet—that the researcher’s involvement was limited to the study period. When this period ended, they were reminded of this, and a closing conversation was held or at least offered. This framing of the relationship allowed participants to appreciate our presence without false expectations: They indicated that the extra company was not experienced as a burden, and in the consultation room the researcher’s observational, non-participatory role and peripheral seating often meant they were hardly aware of our presence, while still valuing our sympathetic ear (Dickson-Swift et al., 2007).

## Results

In this section, the phenomenon of uncertainty as it appears in hospital practice surrounding outpatient care for people with RRMS is described. Excerpts from field notes are included to ground the results empirically in the data (Finlay, 2011, p. 231). These excerpts are our first-person narratives (the “I” in the text). The numbers (Pxx-x) in the text and at the end of each excerpt refer to the participant being shadowed, followed by a number indicating the specific hospital appointment during which that observation was made (Table 1).

### Spheres of uncertainty

**Fig. 1:**
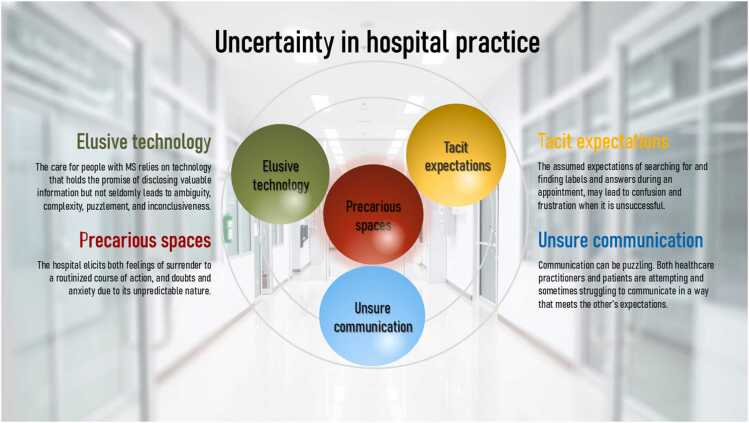
Four spheres of uncertainty in hospital practice surrounding the care for people with RRMS.

Uncertainty is conceptualized as occurring within different “spheres,” understood as abstract entities situated in different contexts. The image of spheres highlights their varied and sometimes opposing (sur)faces, which, like the Earth, shift when rotated around their axis. This study identified four such spheres of uncertainty (Fig. 1): *precarious spaces*, *elusive technology*, *hidden expectations*, and *unsure communication*. Each sphere can either amplify or diminish feelings of doubt, confusion, restlessness, or anxiety among participants and healthcare providers.

#### Precarious spaces

A visit to the hospital includes the unfolding of particular events such as the waiting for an indeterminate period of time or a phone call interrupting the conversation between doctor and patient. These events appear to be a routinized, yet unpredictable, part of hospital organization and the day-to-day conduct of medical personnel. The expected, albeit sudden and erratic, course of action or rules may instill feelings of doubt, insecurity, or powerlessness in participants.

Participants usually spend some time in the waiting room prior to a hospital appointment, varying from two minutes to over an hour. Amidst other people with unknown or suspected neurological disorders, they wait for their name to be called, prone to questions popping up, mind wandering, and nerves increasing. Seeing someone walk past them with great difficulty makes them wonder what their own future will look like. Does the person sitting next to them in a wheelchair have MS as well? When the time of their appointment passes, they might wonder whether they got the time wrong or whether the doctor is just running late. If the doctor is running late, will there be enough time for their questions, or should they try not to take up too much time? Does their own schedule allow for such a delay? What was the purpose of the appointment, anyway? Sometimes, participants are not sure. Other times, they know they will receive the results of their latest MRI scan. The longer they have to wait, the more anxious they get over different and increasingly worsening scenarios. The following excerpt illustrates how routine delays and the ambiguous signals in a waiting room can heighten participants’ sense of uncertainty, fueling both speculation about the cause and concern about the consequences for their appointment:

After talking about holidays in times of COVID, we sit and wait in silence. Suddenly, Michael’s wife laughs and says: “I suppose the neurologist is already behind on schedule!” We support her suspicions and collectively disapprove of the fact that the neurologist is late at the beginning of the day. At some point, Michael and his wife look up as somebody passes through the hallway. It is a man wearing a long white coat who enters a room close to where we’re sitting. “It’s doctor [name],” Michael urgently whispers to me. The neurologist doesn’t acknowledge our presence and shuts the door behind him. It is already 15 minutes past the appointment time and at least another 5 minutes go by before the neurologist comes out of his room again. In the meantime, Michael’s wife and I share an occasional meaningful look as I sense an increasing nervousness with them. (P01–1)

As soon as a participant’s name is called, the waiting is over, and the actual appointment can commence. The consultation room is, on the one hand, a private and secure place, isolated from the rest of the hospital once the door is shut. On the other hand, even this safe space has its unpredictable intrusions and interruptions, such as a phone ringing in the middle of a detailed account of participants' complaints. Participants often stop speaking when the neurologist answers the phone unannounced and converses slightly hushed about another patient:

Abigail asks if the neurologist can confirm that she has the relapsing-remitting type. “This is the first time I can give you more clarity on that,” the neurologist admits, “because you’ve just told me you’ve noticed an improvement in your complaints and when someone….” She is interrupted by the ringing of a phone in the breast pocket of her coat. “Sorry about this,” the neurologist apologizes as she reaches for the phone to answer it. A short and hushed conversation follows while Abigail waits in silence. The neurologist talks about a resuscitation, and when she hangs up the phone, she immediately continues her explanation on the MS subtype. [A few moments later] “No, when it comes to the medication you….” The neurologist is interrupted again by the ringing of a phone. She instantly answers it without an apology or announcement this time. Seemingly bothered, the neurologist asks the person on the other end of the line to contact someone else because she is in the middle of a consultation that is constantly interrupted by phone calls “which is disturbing for the lady in front of me, I think.” (P11–1)

At other times, a knock on the door is followed by a person in white clothes entering the room, either to ask the neurologist a question or to look for a specific object, in which case they open a cabinet or grab an information leaflet and leave the room again without saying a word. Even though a phone call often ends as abruptly as it started, and the person entering the room tries to act like a fly on the wall, the atmosphere is changed, and a feeling of certain and undivided attention is eroded.

The COVID pandemic with its accompanying changeable restrictions imposed another challenge: Is it safe to be in the hospital at all, let alone in a small hospital room with multiple people? The neurologist disinfecting their hands repeatedly might be taken as a bad omen. One participant worried if he could manage having to wear a face mask the whole time, because it gave him trouble breathing. At some point, participants were allowed to come to the hospital, but not to bring anyone with them. This led to the exceptional situation where the researcher was allowed to accompany participants, but their partner or family member was not. Lacking their familiar companion, some participants expressed feeling insecure about remembering all the questions to ask the neurologist and their ability to process and report back on the outcome of the appointment.

#### Elusive technology

Technology is frequently employed by hospital personnel and relied upon by participants. Computers are regularly used in the consultation room. Instead of facing their patients throughout the appointment, healthcare practitioners are often turned towards a computer screen, entering information and updating the patient’s medical record. Furthermore, the care for people with RRMS largely depends on the results of examinations, tests, and scans. In effect, every step forward appears to be technologically mediated.

Of the different technological tools, MRI scans seem to occupy the most crucial role in clinical decisions and the medical follow-up of people with RRMS. A scan is made at periodic intervals, varying from a few months to one or more years, depending on medication use, disease course, and hospital policy. It holds the promise, for both doctor and patient, of learning something about past, present, and predicted future disease course. An MRI demonstrates the unique ability to “look inside one’s head” (P05–2) and make the MS visible in the form of white lesions in the brain and/or spinal cord. The scan seems to legitimize participants and their disease, but at the same time reduces them to a magnetically generated image.

If a scan shows no new lesions, it could mean that the MS is stable, possibly due to medication use. If a scan does show new lesions, the opposite could be true, and there might be therapeutic implications. Although these outcomes seem relatively straightforward in theory, the reality proves to be more complex, as the excerpt below demonstrates. Even when results are presented as largely positive, the interpretive complexity of MRI findings can create additional layers of uncertainty, leaving key questions unresolved:

Previously visible lesions significantly reduced in size, but a new lesion can also be identified, albeit a very small one. So small, that the neurologist hadn’t noticed the lesion himself, but read about it in the radiologist’s report. So small, that it’s unlikely to cause any complaints, although that’s the case for most cerebral lesions. The neurologist assures Nicole that the results are 90 % positive. “So I have a relapse?,” she wonders. “No, a lesion lighting up from the contrast fluid,” the neurologist responds. The highlighting of the lesion is a sign of inflammation, but it doesn’t tell when the inflammation took place. Has the MS been active recently? Or before she started medication? Does it mean then that the medication isn’t effective enough? Should she consider switching medication and if so, to which one? These are all questions running through Nicole’s mind, as she shares with me after the appointment. (P05–2)

If a scan shows no new lesions, but a participant did experience complaints indicative of an exacerbation prior to the scan, there might still be reason for changing the medication. Instead of relying on the MRI for therapeutical decisions, neurologist and patient now have to base their choice on other, less clear-cut considerations. Some neurologists try to explain the occasional “mismatch” of experiencing new complaints without new lesions showing up on the MRI, further complicating its interpretation and value. Furthermore, a stable MRI scan cannot satisfy a participant who has not been feeling stable, but has instead been struggling with persistent complaints–as if they cannot reconcile the MRI results with their own experiences.

Despite its intricate complexity, the MRI remains an important part of a medical appointment. Images are extensively shown and explained to participants, even if they do not fully comprehend them. One could wonder whether the elaborate explanation mostly yields to the demands of a patient or whether it might also stem from the interests of a neurologist. The excerpt below captures how the detailed visual explanation of MRI images—while intended to inform and reassure—can also overwhelm participants, highlighting the gap between clinical interpretation and patient understanding:

The neurologist turns towards her computer as she explains that she will show the MRI images. She tries to demonstrate from what perspective the images are made by tilting her head backwards. “Imagine lying on your back looking up while we are looking at your head from your feet. This is your upper jaw.” She points to a white structure. “And, in a second, we will see your ears and nose.” Ava and her partner laugh as the neurologist scrolls through the images and what is supposed to be Ava’s nose. “This is your brainstem and we are now moving up towards the little brain.” We are looking at changing images of black, gray, and white curved and winding shapes. The neurologist explicates how a certain area of the brain (“juxtacortical”) is of particular relevance to MS, but there are no lesions to be discovered there. There are, however, lesions in other parts of the brain. “Here you see a small lesion, here we see a small lesion, and here a small lesion.” As soon as the neurologist uses the word “lesion,” Ava and her partner throw each other a more serious look. “These are all extremely small lesions,” the neurologist seems to want to reassure Ava, “and we see no differences compared to an earlier scan.” When we talk about the appointment afterwards, Ava shares with me how the elaborate clarification given by the neurologist on the MRI results is somewhat overwhelming and confusing. “Don’t get me wrong, I appreciate seeing the brain pictures, but I don’t understand what I’m looking at at all!” (P12–3)

Here, the nervous anticipation that led up to the MRI results is replaced by considerable relief in the case of a favorable outcome even if, in some cases, the scan does not carry crucial implications because it was made to establish a “baseline” after starting medication use. Neurologists often discuss the results at the beginning of an appointment if they sense a participant is anxious. Participants take pictures or make recordings to share with their loved ones at home. *“*It’s different if I can show it rather than just tell them about it*”* one participant said (P05–2). The sense of relief is, however, fleeting, due to the prospect of a new MRI in the future. Furthermore, not all participants are eager to have the status of their disease visualized and objectified by an MRI because of its possible implications and would rather avoid it altogether.

Diagnostic tools other than MRI also offer participants the prospect of valuable insights and certainty, for instance, through regular blood tests to check for abnormalities caused by medication use. Such tests can provide reassurance by ruling out alternative causes for symptoms, or by confirming that treatment is working as intended. For example, a test for iron deficiency may help explain a participant’s fatigue, while testing for COVID-antibodies can guide decisions about vaccination. In other cases, a blood test can validate or eliminate a specific medication option, such as verifying prior infection with chickenpox before starting medication. Although hospitals increasingly allow patients to access test results online, which may shorten the anxious waiting period before an appointment, this immediacy can also introduce new uncertainty when results are difficult to interpret:

Margaret verifies with the neurologist that she no longer receives the customary phone call after a blood test. The neurologist confirms, but suggests she can always contact the MS nurse herself if she wants to discuss the results. Margaret goes on to explain the reason for her question: “When I study the results at home, I notice the level of all the good things falling and of the bad things rising. Of course, I’m not the doctor, I’m not the expert on this, so my own explanation is not completely accurate. But as a patient, I don’t feel reassured by certain numbers going down. What you said earlier, about the lymphocytes going down being a good thing, I did not expect that!” The neurologist stresses again how the medication is supposed to suppress the lymphocytes, and the minimum level of 0.2 is closely monitored through the blood tests. After the appointment, Margaret still seems a little discontented and worried: “I didn’t know the lymphocyte levels were expected to drop. It is 0.3 now, but it might as well be 0.2 soon. You see all these lab values—AST, ALT—but have to find out for yourself what they mean.” (P15–1)

This interaction shows how access to test results from home, while intended to empower and inform, can also introduce new uncertainties when patients must interpret unfamiliar numbers without immediate professional guidance.

#### Tacit expectations

Certain implicit assumptions seem to underly the setup of a medical consultation, such as the image of the neurologist as an omniscient being, especially when it comes to MS. Participants save up or write down their questions on a piece of paper or in their phone prior to an appointment, eagerly anticipating their rare meeting with the neurologist. Questions are of practical or predictive nature and are not always guaranteed a certain or straightforward answer. Questions also concern the planning of future MRI scans, the cause of certain complaints, the necessity of a COVID vaccination, the safety of complementary medicine, and planning of a pregnancy. Ample time for questions is created by the neurologist or taken up by a participant. A reassuring answer from a healthcare professional seems to erase many doubts or uncertainties; however, missing or unclear answers by the end of an appointment leave participants feeling somewhat disappointed and puzzled.

While face-to-face with their doctor, participants also tend to report back in meticulous detail on physical changes or other specific incidents related to their health. They elaborate on the date, time, and place they first noticed an altered sensation, where and how they felt it in their bodies, what they were doing at that moment, etc., as if to make sure the neurologist obtains all the necessary pieces to solve the puzzle, not knowing exactly what pieces they need and how best to convey their concerns. Sometimes, participants add their own interpretation or possible explanation of the incident. They might be showing the neurologist that they have contemplated the occurrences, attempting to nudge the response in a certain direction, or softening the blow for themselves in case the neurologist expresses worry.

Although not always explicitly asked, the key questions underlying such elaborate accounts seem to be “Is this linked to MS?” or “What is the explanation?” Once a participant finishes their story, at times interrupted with probing questions or cut off altogether, the neurologist often provides these implicit questions with an answer:

At some point, Dylan presents his own hypothesis and wonders whether his own worry over the dizziness caused his other complaints. The neurologist doesn’t react immediately, but rather, asks him one final question before arriving at what seems to be her own conclusion. She stops typing and says, “Considering everything you’ve said, it is likely that physical exertion caused the loss of motor function. We call it ‘vasovagal’ or loss of consciousness due to a drop in blood pressure. It is a common phenomenon in young people when they strain themselves too much, especially under hot circumstances and if they also change posture.” A migraine or epileptic episode is mentioned, but instantly ruled out. Finally, she adds that his MS didn’t cause the episode: “It was so short, it is completely unrelated to MS, that is a sure thing.” (P10–3)

In this interaction, the exchange reflects an almost reflexive dynamic: Participants’ detailed accounts implicitly seek to determine whether a symptom is linked to MS, while the neurologist responds with an equally immediate ruling on its (un)relatedness to the disease.

If the complaints are not linked to MS, this is often stressed in a reassuring manner. The reassurance elicits relief, but at the same time, ambiguously undermines the burden the complaints might nevertheless place on the one experiencing them. If the complaints are linked to MS, they are generally attributed to existing lesions flaring up due to stress, considerable exertion, or an infection, instead of to a new exacerbation. Participants are informed about basic underlying physiological mechanisms, sternly warned against stepping over their own energetic boundaries, or referred for diagnostic blood or urine tests (to show a possibly treatable infection). Occasionally, the option of physical, occupational, or rehabilitation therapy is discussed, or an educational folder is handed out.

However, the answers provided to these or other questions a participant may have, are not always black and white. Participants are left wondering how to define and tackle their concerns and complaints. Some neurologists offer practical advice, suggest specific treatments, or try to convince participants to make certain adjustments in their daily lives to address the problems. Other neurologists are inclined to refer their patient to another specialist or suggest they make an appointment with their general practitioner. Some participants are pleased with the extensive exploration. In some, it prompts worry, and others feel frustrated at the lack of definitive answers. The scene below highlights how, when definitive answers are lacking, the exchange can move into a space of tentative explanations and conditional suggestions, often leaving participants with lingering uncertainty despite having voiced their concerns:

“Do you have any other questions for me?” the neurologist wants to know, and Sarah immediately mentions her headache. “I still suffer from headaches every now and then, basically since the exacerbation, but not as severe as it was before. I’m not sure what is causing it?” she ends with a question. There is a short silence before the neurologist replies. “That is a good question with a tricky answer, in my opinion. The scan doesn’t show any abnormalities that would explain your headache, limiting our treatment options. Medication usually offers little relief. You can always try physical or postural therapy,” the neurologist proposes, leaving Sarah a little disheartened. “And is it normal to be so easily overstimulated by external stimuli after an exacerbation?” Sarah additionally wants to know. “It is impossible to say what is normal when it comes to MS,” the neurologist immediately discloses. “Many symptoms *can* be attributable to MS,” he stresses. “Hypersensitivity *can* be a residual complaint, but not everyone with MS has to experience this. It is hard for me to determine.” Sarah’s reply with a soft tone of voice seems to unveil her disappointment. (P07–1)

Another example of the tacit assumptions underlying the setup of a medical consultation is the supposition of patients being able and willing to answer any medical question. Part of almost every medical appointment is the neurologist asking probing questions in response to information shared by the participant or running through a checklist of predefined questions. Seemingly dictated by what needs to be documented in a participant’s medical record, the neurologist addresses a series of closed questions, either to learn more about complaints expressed by the patient or to gain insight into various general domains such as cognition, mood, sensory and motor function of limbs, sexual activity, and bowel movement, conferred in layman’s terms. The interaction between neurologist and patient suddenly resembles an interview, consisting of a quick back and forth of questions and answers. The participant saying little more than “Yes” or “No” and the neurologist typing up a summary of the response into the computer.

Sometimes, the questions require a more elaborate answer, and participants struggle to remember specific details or respond to a question they did not see coming:

“How is your vision now, is it normal again?” the neurologist asks, and Matthew replies with just a “Yes.” “No double vision?” she inquires, and he confirms. The neurologist then informs about his facial sensations and Matthew reacts positively. “The sensation in your arms?” the neurologist continues, not formulating a complete sentence this time. Matthew explains that his right arm did bother him for a while, but he thinks this is less now. Although the conversation has gradually taken on a more interrogatory tone, Matthew openly responds to all questions. His answers may be short and his use of the word “less” somewhat confusing, but the neurologist seems content with and to understand his responses. “How would you say peeing is going?” the neurologist asks a little out of the blue. “Fine” Matthew briefly states before adding he did struggle in the past. The neurologist is curious about what he was struggling with specifically. Matthew explains he could suddenly feel a strong urge that led to difficulties with holding it in. “And defecation?” The back and forth of questions and answers continues, only occasionally interrupted by the neurologist typing on her computer. “Are there any problems sexually, erectile dysfunctions?” Without batting an eye, Matthew once again says this has been less, but has returned to normal. (P17–1)

This excerpt shows how a shift toward rapid-fire questioning can transform the consultation into an interview-like exchange, with varying effects on participants’ comfort and recall. Furthermore, medical-technical thoroughness and box-ticking take center stage, potentially diverting attention from the participant’s main concerns.

At times, a physical examination is carried out as well, the questions turning into verbal and tactile instructions. Participants are asked to remove a piece of clothing, lie down, pull their knees up, push the neurologist’s hand away, follow a moving finger with their eyes, walk on their toes or in a straight line, and get dressed again. Findings are compared to previous physical examinations and a conclusion (better, stable, worsened) is drawn. In the end, the neurologist is usually the one to indicate when the appointment is over. Often preceded or followed by a “Do you have any more questions for me?” the neurologist highlights the most important outcomes of the consultation and briefly summarizes future planned proceedings such as when the next appointment, test, or scan will be and any other agreements made during their consultation.

#### Unsure communication

Regardless of the topic (MRI results) or form (back and forth of questions) of the conversation, communication itself can be puzzling—a continuous balancing act of when to speak, what language and words to use, and how to be responsive to the other person’s needs. Specific words used casually by a healthcare practitioner may carry an elevated meaning for participants, such as for the participant whose diagnosis of clinically isolated syndrome (often a precursor to MS) was recently changed to multiple sclerosis and who now has an appointment with the specialized MS nurse to discuss medication options. Halfway through the conversation, the specialized MS nurse starts to use the word “disability” a lot, explaining how the disease could progressively damage this participant’s nervous system in the future. The participant sits there, listening in silence, seemingly unshaken, but confides in me afterwards that this “harsh” word had “startled” her (P12–1). Another example is the use of the word “fatal” when describing a side effect of a therapy (P14–2). Words can fuel uncertainty, but can likewise reduce it. Hearing the neurologist describe the results of an MRI scan as “good” can be immensely relieving (P16–1).

Furthermore, words can be complex or overly numerous. Complexity arises from medical jargon that neurologists use, possibly unintentionally, but nevertheless, with frequency. The implication of words such as “surrogate marker” (P01–2), “lhermitte’s phenomenon” (P02–1)*, “*first-line therapy” (P04–1), “lymphocytes” (P08–1), “methylprednisolone” (P10–1), “juxtacortical” (P12–3), “transmural” (P14–2)*,* “real world data” (P16–1), “punctiform” (P17–2), and “burden of disease” (P17–3) may not be obvious to everyone. At other times, neurologists go through great lengths to put medical matters in layman’s terms, for instance, when explaining why an MRI of the spinal cord is challenging to read. The neurologist might compare the spinal cord to “an empty kitchen roll filled with water and a bunch of spaghetti strings” (P16–1). Finding the right balance between medical jargon and everyday language is a delicate task, and it varies for every patient. However, even well-intended, detailed explanations can become difficult to comprehend. In a physician’s attempt to be thorough and transparent, participants find themselves lost in translation:

The neurologist informs Dylan of the different steps that need to be followed before he can start with the medication [Zeposia]. “First, we want to make a recording of your heart to examine if it beats regularly and fast enough.” Dylan proclaims he had already read about this in the information leaflet. The neurologist continues to explain that he should check with the clinic’s reception after their appointment to see if the ECG can be done today. “We will also draw some blood, and I would like the dermatologist to check your skin. Zeposia can sometimes cause malignant degeneration, as we call it, of previous lesions which makes it important for a dermatologist to regularly examine your skin. The specialized MS-nurses will monitor when it is time for a new check-up, but for now, we would like to determine if there are any suspicious lesions beforehand in which case Zeposia might not be the best choice.” After the appointment, I wait for Dylan to make the necessary arrangements at the reception. When he returns to me, he refers to the different steps as “the whole shebang” and admits how he had trouble understanding the explanation of the neurologist. Usually, his girlfriend can translate it to him in layman’s terms, but she wasn’t allowed to join us today. It turns out he doesn’t know what a “dermatologist” is, even though he did not ask the neurologist for a clarification during the appointment. (P10–1)

Participants can also struggle with a physician’s answer or explanation, simply because they do not understand or accept it. In such cases, it is not that the neurologist’s account is too complex or elaborate, but rather, that it does not match a participant’s own ideas, logic, or feelings.

In the end, it seems that both healthcare practitioners and participants are attempting and sometimes struggling to communicate in a way that meets the other’s expectations. Participants may wonder whether they should continue speaking when the neurologist starts typing, or whether they should wait until the typing is finished before asking a follow-up question. They may question whether the conversation is over once the neurologist pushes back their chair and gets to their feet*.* They may also struggle to get their message across when interrupted or when the responses they receive are unsatisfactory. Some participants choose to interrupt in turn, repeat themselves, or emphasize the strain their symptoms place on them (“I almost died of the pain” [P07–3])? Others adopt medical jargon (“oligoclonal” [P03–1], “terminology” [P12–2]) to present themselves as knowledgeable and to be taken seriously. The following excerpt illustrates how one participant struggles to make herself heard and understood:

Patricia immediately reacts to the announcement she probably has the relapsing-remitting subtype of MS with “Relapsing-remitting usually develops in younger people!” As she was 60 years of age when she was diagnosed with MS, her response suggests she has doubts about the established subtype. The neurologist tackles her objection by explaining that it is unknown when she has had her first exacerbation, possibly a long time ago. Patricia still doesn’t seem to accept the diagnosis. “MS has many different symptoms, and I’m being told that my complaints, such as double vision, are not related to the MS. The same goes for my back pain. This has gotten worse, but no one wants to examine my back. It is very confusing to me that the only response I get is ‘not MS related’.” She sounds a little agitated and uses the words “confusing” and “not MS related” a lot. It is not immediately clear to the neurologist what her concern is as he asks her for clarification several times. (P03–1)

For neurologists, the choice of words is entangled with the assumed wishes of a patient who may need reassurance, which can be difficult to provide when the facts are unclear or unfavorable*.* Others may primarily long for an honest answer, even when it is difficult to hear. Neurologists also face the challenge of disclosing the limits of their own knowledge and of medicine in general to a person with an insatiable need for information and clarity. In addition, neurologists must sometimes convey the necessity of an expedited scan to a person who rather shies away from objective assessments. Besides the inherent (medical) uncertainty of the disease, doctors seem to struggle with uncertainty about how best to approach a patient. Within the boundaries of their profession, they have some leeway to highlight the chances of an upward progression, to leave unlikely, but unfavorable, odds unsaid, or to make predictions of the future based on clinical experience. At times, doctors might not be able to satisfy the needs of a patient, which can lead to frustration and confusion on both sides.

The neurologist tries to summarize everything they have discussed so far. “The scan shows no lesions lighting up. Your body is cleaning up the inflammation. The amount of residual damage can be established in another one or two years. As for the second vaccination, I would say you are now sufficiently protected, but the production of new antibodies in response to the vaccination might be less than expected, due to the Ocrelizumab.” Sophie inquires about the safety of a second vaccination, and the neurologist states: “Of course, we can never be completely sure, so in the end, it’s a matter of assessing the risks.” Sophie replies: “I find that very difficult. What if you would do that for me?” The neurologist confesses it is difficult for him as well: “There is no literature to base a decision on. You are the only person I know who has reacted so adversely to the first vaccination.” He then confides in her that, if he were in her shoes, he would probably be hesitant as well. “But I have to be honest with you, I just cannot know for sure.” Sophie somewhat reluctantly says she appreciates his honesty. (P02–1)

This excerpt illustrates how unsure communication emerges when mismatched expectations, uncertainty about conversational cues, and differing interpretations of symptoms intersect with the neurologist’s own uncertainty over how to respond—balancing honesty, reassurance, and the limits of medical knowledge and leaving both parties striving, yet sometimes struggling, to reach mutual understanding.

## Discussion

This study portrays uncertainty in hospital practice surrounding outpatient care for people with RRMS as manifesting in different spheres. The four spheres pertain to hospital organization, the use of technology, and the expectations of and communication between patient and healthcare provider. In the following sections, the findings will be reflected on in relation to existing literature. The implications of the findings for healthcare practice will be discussed as well as the limitations and strengths of the study.

### Reflections on the findings

First, uncertainty manifests in the hospital arising from its routinized, yet unpredictable, sights, occurences, and organization. As Hans Westerveld (2023) argues in his dissertation on the meaning of the hospital building, hospitals are set up as a machine designed around the care process, in order to achieve both (technologically mediated) safety and the (aesthetic) security of a healing environment. The *precarious spaces* of this study extend beyond the hospital’s architecture to include the ways in which (uncertainty in) the hospital is experienced, positioning the hospital as a “lived space” (Van Manen, 2016). Finlay (2011, p. 20) illustrates this “lifeworld existential” by how a dark alley may be perceived as threatening or a house can feel cozy.

There is scant research on the hospital as a lived space. Faux-Nightingale et al. (2024) depict the difference between the meaning of a hospital corridor prior to and during the COVID-19 pandemic. Prior to the pandemic, it was perceived as a social and symbolic space for collective sensemaking. Later, the “COVID-19 empty corridor” was a haunting place. The lived experiences of stroke survivors admitted to an acute stroke unit are captured by Suddick et al. (2021) as both a safe haven and a transitional space. Van der Meide et al. (2015) frame the meaning of hospitalization for older patients as feeling like an outsider left in uncertainty. These studies demonstrate that hospitals can be experienced in diverse ways, depending on the broader context (e.g., a pandemic), the purpose of a specific space (e.g., treating stroke patients), and patient characteristics (e.g., age).

Connecting the uncertainty experienced by people with RRMS and their healthcare providers to the hospital context—particularly waiting and consultation rooms—understood as a lived space, represents a novel contribution. It has revealed that the waiting room of a hospital can elicit feelings of anticipation, insecurity, or anxiety. These feelings seem connected to the (unknown timing of the) opening of a door by the neurologist, the prospect of entering the consultation room, and the conceivable breaking of good or bad news. Once in the consultation room, the shutting of the door may instill a feeling of intimacy and safety. The intimacy of the undivided attention of a neurologist and the safety of an enclosed room are easily lost when a phone rings or the door is promptly opened from the outside. Within the context of this study, therefore, the hospital as lived space could be characterized as one that elicits a feeling of surrender to a routinized course of action, alongside doubts and anxiety due to its unpredictable nature.

Second, the results show that technological reliance characterizes not only the care provided to people with RRMS, but also the experiences of people with RRMS themselves—technology that holds the promise of disclosing valuable information, but often leads to ambiguity, complexity, puzzlement, and inconclusiveness. Clinging to diagnostic tests for answers might be a short-term solution, as it only postpones the decisions that have to be made eventually. Complexity is especially prevalent in case of the MRI. Though assigned significant agency, its outcomes are not always black and white, and it is up to human interpretation—first a radiologist and then the neurologist—to establish its implications. Patients usually lack the knowledge doctors have to interpret the technology, making *technology elusive* in the mutual attempt between doctor and patient to understand its implications.

Although technological progress has improved health and well-being, it is not without its dangers (Galvin & Todres, 2013, p. 9). Heidegger warned against submitting to the totalizing spirit of technology and the presumption that its application always constitues an improvement (Finlay, 2011, p. 51). A focus on the promising, informative nature of the MRI (Filippi et al., 2016, Wattjes et al., 2015) and other biomarkers (Ziemssen et al., 2019), is shown in the ample studies that aim to increase their precision and diagnostic value for MS care. This study presents a more nuanced image of technology, specifically the MRI which serves a prominent role in MS care, showing both its promises and perils.

Third, *tacit assumptions* and *unsure communication* pervade the encounter between patient and the healthcare professional. The conversation seems to unfold as if implicitly scripted, with both parties constantly countering each other’s requests, posing questions, taking the lead, or falling silent. The subject matter is mainly medical-technical, striving towards ticking boxes and putting forward explanations. It is as if thoroughness guarantees certainty, yet it might also divert attention from a patient’s main concerns. Searching for labels and answers during an appointment may lead to confusion and frustration when unsuccesful. In addition, communication itself can be puzzling. Words can bear considerable (positive or negative) meaning and be complex to patients who, in turn, attempt to adopt the medical jargon. Both healthcare practitioners and patients seem to, at times, struggle to communicate in a way that meets the other’s expectations: patients by considering when to speak, how to get their message across, or what words to use, and healthcare practitioners by seeking a balance between providing medically relevant information and being responsive to a patient’s supposed wishes.

Some of these findings can be mirrored to the uncertainty in interpersonal communication described by Berger and Calabrese (1975) in their “uncertainty reduction theory” (URT). According to Berger and Calabrese (1975), individuals experience uncertainty due to unknown personal attributes and unpredictable behavior. For the interaction and relationship to develop, individuals engage in uncertainty reduction. When communication is unsure—and, according to the URT, uncertainty levels are high—the interaction partners tend to follow implicit and explicit norms or rules, display information seeking behavior, share limited intimate information or do so in a non-reciprocal manner, and establish little verbal and non-verbal communication. Several of these strategies seem inherent in the doctor-patient relationship, as the physician will always have some “information power” (Berger & Calabrese, 1975, p. 105) over the patient, and healthcare seems to be fixed on finding the right answer through collection of information (Simpkin & Schwartzstein, 2016).

### Recommendations for healthcare practice

This study shows that uncertainty appears to be a widespread, but masked phenomenon. Buetow (2011) corroborates the tacit nature of uncertainty in healthcare, acknowledging a patient’s desire for answers from their healthcare provider and a clinician’s inclination towards “ordering excessive clinical investigations” in order to satisfy this desire, as it can be stressful to admit to uncertainty. When uncertainty is acknowledged, it tends to be seen as a problem that needs to be reduced (Berger and Calabrese, 1975, Buetow, 2011). An oversimplified recommendation for healthcare practice would be to unveil uncertainty through examining and debating the course of action at the outpatient clinic, as well as the promises and perils of technology, and through metacommunication with patients*—*that is, explicitly talking about expectations, assumptions, and uncertainties in the communicative process itself. Hospital practice is, however, a complex institution with time constraints and personnel shortages. As such, it would be unfair to imply that this recommendation is not already acted on by healthcare practitioners.

Additional suggestions can be found in the work of other authors, some of them already mentioned. Buetow (2011) proposes that uncertainty needs to be viewed differently from the dominant risk management approach. Uncertainty in itself has no fixed meaning, but people are “relationally disposed…to perceive uncertainty in their particular social situation, on the basis of their lived experience” (Buetow, 2011, p. 874). People can interpret uncertainty as a problem, but the ability to tolerate uncertainty can be a virtue that allows for uncertainty to be a potentially positive force (Buetow, 2011). Galvin and Todres (2013) present eight humanizing values (e.g. insiderness, sense of place, and embodiment) with which they imply that the statistical, technical, problem solving aspects of care should not overshadow the experiences, subjectivity, and uniqueness of the patient (Galvin & Todres, 2013, p. 11–19). Goossensen (2011) also warns against reduction or the process in which healthcare providers reduce the complexity of a patient’s issues to diagnostic terms and categories, running the risk of insufficient attunement to a patient’s expectations.

The previously discussed URT by Berger and Calabrese (1975) offers particularly practical guidance. As Berger and Calabrese explain, it is through non-verbal communication (eye contact, head nods, head and arm gestures, etc.) that uncertainty can be diminished (Berger & Calabrese, 1975, p. 102). The fact that healthcare practitioners are often turned towards a computer screen, losing the opportunity to respond to a patient in a supportive non-verbal way, raises questions on the increasing use of computers in the consultation room.

Finally, we wish to underscore the importance of a “sense of place” (Galvin & Todres, 2013, p. 17). As suggested by Galvin and Todres, more attention needs to be put toward the quality of space in healthcare environments, making it more conducive to privacy, dignity, homeliness, and hopefulness (Galvin & Todres, 2013, p. 17).

### Limitations and strengths

This study constitutes a unique design of a longitudinal follow-up of participants in their first two years of living with a chronic disease. Shadowing them in the hospital allowed for a close observation of the phenomenon of uncertainty as it unfolds in practice, rather than reflecting on it through interviews. Although most participants reported that, in hindsight, they often forgot there was another person in the consultation room, it is not inconceivable that the researcher’s presence did affect the unfolding of the appointment. Occasionally, a participant noticed a change in the neurologist compared to encounters without the researcher present, such as giving more thorough or elaborate explanations and taking more time. We cannot account for the appointments we did not attend, but the high number of observations (39) should cancel out significant variations. Furthermore, in contrast to quantitative research, the goal of qualitative research is not to prove causality or generate statistically generalizable outcomes. Instead, the goal is to gain insight into the human condition and to resonantly describe the lived world of everyday experience (Finlay, 2011, p. 10). Finally, the perspective of healthcare practitioners is not explicitly addressed. Although we shadowed patients, as they are the ones living with an uncertain disease, the findings also reveal that healthcare practitioners face challenges, including in communication characterized by uncertainty. Thompson and colleagues (2022) report similar issues of stress, anxiety, and burden of care that healthcare providers experience. Future research could therefore include interviews with or additional shadowing of healthcare practitioners.

## Conclusions

This study explored the phenomenon of uncertainty as it appears in hospital practice surrounding outpatient care for people with RRMS. The results show that uncertainty manifests in four different spheres: 1) precarious spaces, 2) elusive technology, 3) hidden expectations, and 4) unsure communication. The spheres never seem to fully surface; rather, they operate implicitly, shaping interactions and experiences without being openly acknowledged. In this sense, uncertainty can be seen as a masked phenomenon. These findings imply the need for an “unveiling” of uncertainty through examining and debating the course of action at the outpatient clinic, as well as considering the promises and perils of technology and the need to metacommunicate with patients. Answers or reassurance from a healthcare professional seem to erase many doubts and uncertainty. Nevertheless, a view of uncertainty different from the dominant risk management approach would improve situations where uncertainty is irreducible—which, according to different authors, is inevitable in healthcare. This way, the statistical, technical, and problem-solving aspects of care are less likely to overshadow the experiences, subjectivity, and uniqueness of a patient. Finally, more attention could be paid to the quality of space in healthcare environments, including the increasing use of computers, making it more conducive to privacy, dignity, homeliness, and hopefulness.

## CRediT authorship contribution statement

**Erwin L.J. Hoogervorst:** Writing – review & editing, Validation, Resources, Project administration. **Inge A.M. van Nistelrooij:** Writing – review & editing, Validation, Supervision, Methodology, Formal analysis, Conceptualization. **Eva C. van Reenen:** Writing – original draft, Visualization, Project administration, Methodology, Investigation, Funding acquisition, Formal analysis, Conceptualization. **Alistair R. Niemeijer:** Writing – review & editing, Validation, Supervision, Methodology, Formal analysis, Conceptualization. **Leo H. Visser:** Writing – review & editing, Validation, Supervision, Resources, Funding acquisition, Formal analysis, Conceptualization. **Janet W.K. de Beukelaar:** Writing – review & editing, Validation, Resources, Project administration. **Bob W. van Oosten:** Writing – review & editing, Validation, Resources, Project administration. **Stephan T.F.M. Frequin:** Writing – review & editing, Validation, Resources, Project administration.

## Funding

This work was supported by the Dutch National MS Foundation [grant number OZ2019-008] and Merck Group [Uncertainty MS fellowship]. The funders had no role in study design, data collection and analysis, decision to publish, or preparation of the manuscript.

## Declaration of Competing Interests

The authors declare that they have no known competing financial interests or personal relationships that could have appeared to influence the work reported in this paper.

## Data Availability

Data cannot be shared publicly due to potentially identifying information and lack of participant consent for sharing pseudonymized data. Data excerpts and other descriptions are presented in the article. Additional data may be available upon request in the DANS repository: https://doi.org/10.17026/SS/7T9L36.

## Footnotes

2 Hereafter, for simplicity, we will refer to ourselves in the plural (e.g., “us” and “we”), although, strictly speaking, we traded off on responsibilities. In other words, we conducted some tasks individually, but overall, worked as a team.
